# Amesh Adalja: taking pandemic preparation seriously

**DOI:** 10.2471/BLT.20.030520

**Published:** 2020-05-01

## Abstract

Amesh Adalja talks to Gary Humphreys about pandemic preparedness and the challenges posed by COVID-19.

Q: How did you come to your focus on pandemics?

A: I read a book called *Level 4: virus hunters* of the CDC just after college. That book had a huge impact on me. I went to medical school wanting to develop a 360° view of the different challenges pandemics present and to develop the skills needed to respond to them. I ended up specializing in infectious diseases as well as emergency medicine and critical care.

Q: Pandemic preparedness is at the core of your work at Johns Hopkins University. How much of that work is focused on coronaviruses?

A: A significant amount. In 2018, for example, I headed up the writing of a comprehensive report on pathogens with pandemic potential, which included a section on coronaviruses and specifically recommended prioritizing improving surveillance of human infections from respiratory-borne RNA viruses – of which SARS-CoV-2 is an example. It’s also worth mentioning that the Johns Hopkins Center for Health Security hosted a table-top exercise in New York in October 2019 that modelled the development and impact of a coronavirus pandemic referred to as Event 201. The exercise was run in collaboration with the World Economic Forum and the Bill and Melinda Gates Foundation. It highlighted areas, such as supply chain logistics, where public-private partnerships can play an important role.

Q: So, would it be fair to say you, and experts like you, saw this pandemic coming?

A: The likelihood of an epidemic like this occurring has been staring us in the face for some time. Coronavirus-related disease epidemics such as SARS (severe acute respiratory syndrome) and MERS (Middle East respiratory syndrome) were both important wake-up calls. There have also been a number of epidemics with zoonotic origins, another SARS-CoV-2 characteristic, including the H1N1 influenza pandemic, and the Ebola and Zika epidemics. Throughout these different epidemics, pandemic preparedness professionals like myself have been doing research, conducting exercises, making policymakers aware of threats, actual and potential, and advising them how to respond.

Q: Given all this foresight and activity, why do you think many countries, including high-income countries, have seemed so unprepared for the event we are now facing?

A: One possible explanation is a general lack of seriousness with regard to threats pandemics pose. The truth is there is always a spike in interest during an emerging infectious disease epidemic, but once the disease recedes the interest goes away. This is as true of policymakers as it is of the media. In some cases, governments have actually reduced pandemic response capacity as a result of this lack of seriousness.

Q: Both the SARS and MERS outbreaks were relatively brief and geographically limited. Do you think this led to complacency with regard to SARS-CoV-2?

A: In the initial stages, I think people were looking at SARS-CoV-2 as something that could be containable and would not have efficient human-to-human transmission, based on observations of the way coronaviruses tend to behave. The four common human coronaviruses display good transmissibility but do not cause serious illness in most patients, while the coronaviruses that cause SARS and MERS, can cause serious illness and have case fatality rates of around 15% and 30% respectively, but they are not very transmissible. Because SARS-CoV-2 looked similar to MERS in some respects, policymakers may have felt SARS-CoV-2 was containable in China. They were wrong. What we – by which I mean the global health community – should have been doing since at least 2003 is developing vaccines against coronavirus-related diseases. Without wanting to understate the size of the challenge, it seems to me there has been ample time to develop such vaccines, some of which may have provided cross-protection against the new coronavirus. Had we done that work we would probably be in a stronger position today.

“In some cases, governments have actually reduced pandemic response capacity.”

Q: How do we incentivize research and development into vaccines for emerging pathogens?

A: This is, of course, a core challenge, and efforts need to focus on mobilizing funds for priority pathogens such as the diseases on WHO’s R&D Blueprint list which currently includes COVID-19, MERS and SARS, among others. Dr Peter Hotez and his team collaborated with scientists at the University of Texas on a SARS vaccine, but they only got as far as animal trials due to a lack of funding.

Q: Do you think the cost of the unprecedented economic bail-out packages being implemented will change minds about the cost of investing in ‘unprofitable’ vaccines?

A: Perhaps. With regard to the broad cost-benefit calculation, people have been conditioned by prior emerging infectious disease outbreaks. Not that the cost of those outbreaks was negligible. The cost to the global economy of the SARS outbreak has been estimated to have been around US$ 40 billion, for example. However, that will pale in comparison to the economic cost of the COVID-19 pandemic. UNCTAD (United Nations Conference on Trade and Development) estimated a loss of US$1 trillion to the global economy in 2020 alone. That level of economic loss may change attitudes. So, certainly investment in vaccines should be prioritised. I think we have to think of pandemic preparedness as part of our national security policy – because that is what is now being threatened by this disease.

Q: Governments have sought to ‘flatten the curve’ of SARS-CoV-2 infection through social distancing measures. Are these effective?

A: There is plenty of evidence that preventing contact between people reduces infection rates. However, keeping people from working entails social and economic costs that can negatively affect population health. Economic shutdowns and the rising unemployment and shortages they entail have been predicted in numerous tabletop exercises. Those exercises have also predicted knock-on health effects of such measures, including increases in deaths from cancer, cardiovascular disease, stroke, mental illness and substance abuse – the combined burden of which may be greater than the morbidity and mortality associated with the virus itself. Right now, one of the things that keeps me up at night is the tipping point at which the benefits of social distancing are outweighed by the costs. I believe that, on balance, it is probably a mistake to shut people away long term. However, it is clear that vulnerable populations, such as older people living in nursing homes should be shielded from everyday life to minimize their chances of infection.

“Just as in the H1N1 epidemic, the poorest are going to be hit hardest.”

Q: Doesn’t easing up on social distancing risk a spike in cases and health systems being overwhelmed?

A: That is clearly the biggest consideration and resources should be committed to expand hospital capacity. In the short-term, governments should work with private partners and other stakeholders to scale up the production of mechanical ventilators and personal protective equipment for health-care workers. We also need to boost the workforce. One way to do this quickly in the USA is to ease state regulations so that nurse practitioners, nurse anaesthetists, physician assistants, paramedics and other relevant staff can take on the tasks currently carried out by physicians. It is also vital that we increase testing in order to better allocate the medical resources we have. Testing will also help individuals infected with the virus to know their status and self-isolate while also providing insights into community disease prevalence.

Q: Many countries, including the USA, have introduced travel bans and border closures. Do such measures help to control spread of SARS-CoV-2?

A:**The literature suggests that travel bans, trade restrictions and the closing of borders have only a limited impact on the spread of disease, especially for a respiratory virus that spreads efficiently. However, politicians are not generally responsive to scientific evidence and are more likely to respond to public pressure. There are many examples of political rather than scientific decisions being taken in the current pandemic.

Q: Some politicians have expressed optimism about the epidemic winding down in the next few months. Is this realistic?

A: There are plenty of indication that we are at the beginning of the pandemic. Many countries have yet to see widespread community transmission, for example, but I think that this is coming. It is a particular concern in populations living in crowded conditions, whether in informal urban developments or in refugee camps. It is going to be very difficult to implement social distancing in any meaningful way in those settings and as a result people living there are going to be more exposed to infection. Some of those people may also be malnourished or have other risk factors. So, I think that just as in the H1N1 epidemic, the poorest are going to be hit hardest.

Q: The Event 201 exercise predicted a maximum 65 million deaths worldwide in the first 18 months for the hypothetical coronavirus pandemic it modelled. What do you project for COVID-19?

A: I don’t think we have enough granularity to know what the numbers will be, but H1N1 infected about 20% of the world’s population within a year and that had population immunity pushing back. We don’t have population immunity to SARS-CoV-2 so we can assume that without effective confinement, between 30 to 50% of the global population will get this over the next year or so. It’s important to remember that most of the COVID-19 cases are going to be mild. I still don’t think we have a good grasp of the case fatality ratio so will hold off on projecting deaths. Also, we need to remember that there may be breakthroughs in terms of antiviral medicines and eventually a vaccine.

Q: Presuming we come through this pandemic, what should we be preparing for next?

A: The next pandemic. There are a number of pathogens that have pandemic potential, as noted in the report we published in 2018. Among those pathogens is the H5N1 avian influenza virus, which has a mortality rate of around 60%. To date the virus has not transmitted from person to person very easily, but that could change. We need to prepare for such events. And we need to take that preparation seriously.

